# FGF21 acting on the noradrenergic nervous system protects against influenza virus infection

**DOI:** 10.1073/pnas.2522045122

**Published:** 2025-09-25

**Authors:** Wei Fan, Yuan Zhang, Laurent Gautron, David G. Thomas, Heather W. Stout-Delgado, Edward J. Schenck, Tadiwanashe Gwatiringa, Kartik N. Rajagopalan, David J. Mangelsdorf, Steven A. Kliewer

**Affiliations:** ^a^Department of Pharmacology, University of Texas Southwestern Medical Center, Dallas, TX 75390; ^b^Center for Hypothalamic Research, Department of Internal Medicine, University of Texas Southwestern Medical Center, Dallas, TX 75390; ^c^Division of Pulmonary and Critical Care Medicine, Department of Medicine, Weill Cornell Medicine, New York, NY 10065; ^d^Division of Pulmonary and Critical Care, Department of Internal Medicine, University of Texas Southwestern Medical Center, Dallas, TX 75390; ^e^HHMI, University of Texas Southwestern Medical Center, Dallas, TX 75390; ^f^Department of Molecular Biology, University of Texas Southwestern Medical Center, Dallas, TX 75390

**Keywords:** FGF21, βKlotho, influenza, energy expenditure, noradrenergic nervous system

## Abstract

The hormone fibroblast growth factor 21 (FGF21) is induced in murine liver in response to both bacterial and viral infection. In this report, we show that FGF21 is induced by infection with influenza virus in both humans and mice. Mice lacking FGF21 had decreased food intake, body weight, and body temperature compared to wild-type mice following influenza virus inoculation, indicating reduced tolerance to the infection. Conversely, pharmacologic administration of FGF21 after viral infection protected mice against these pathologic changes. Pair feeding studies showed that neither the induction of FGF21 nor the hypothermia was secondary to decreased food intake. Notably, mice selectively lacking FGF21’s coreceptor protein, βKlotho, in noradrenergic neurons were also more susceptible to influenza virus infection, including hypothermia. We show that FGF21 acting on noradrenergic neurons, including those in the locus coeruleus region, stimulates energy expenditure and thermogenic gene expression in brown adipose tissue. Our findings reveal an FGF21-regulated neuronal pathway that protects mice against influenza infection and suggest the potential utility of using FGF21 pharmacologically to improve outcomes after influenza infection.

## Significance

Fibroblast growth factor 21 (FGF21) is a liver-derived hormone that signals to the brain to govern several physiologic stresses. In this paper, we show that FGF21 concentrations in blood are increased by influenza virus infection in both humans and mice, and that in mice, FGF21 protects against the consequences of influenza infection, including decreased food intake, body weight, and body temperature. These beneficial effects are mediated by FGF21’s action in noradrenergic neurons and suggest the use of FGF21 in treating the flu.

Fibroblast growth factor 21 (FGF21) is an atypical member of the FGF family that is induced by a wide variety of metabolic stresses and can function through either autocrine or endocrine mechanisms depending on the tissue in which it is made (1–3). FGF21 acts locally when made in tissues including the exocrine pancreas and adipose tissue but is released into the blood to act as a hormone when synthesized in liver. FGF21 acts through a cell-surface receptor composed of two proteins: a conventional FGF receptor, with FGFR1c the preferred isoform, and a coreceptor named βKlotho (KLB). FGFR1c and KLB together form an interface to which FGF21 binds, with the intracellular domain of FGFR1c serving as the signaling effector. The FGFR1c/KLB complex is present in a limited number of tissues, including exocrine pancreas, both white and brown adipose tissue (BAT), and specific regions of the brain (1–3).

Pharmacologically, FGF21 causes weight loss and improves insulin sensitivity and dyslipidemia in obese mice and decreases liver fat and fibrosis in models of metabolic dysfunction-associated steatohepatitis (1–3). In clinical trials, FGF21 derivatives caused modest weight loss, lowered insulin concentrations, and improved dyslipidemia, although the results differed depending on the FGF21 variant. In mice, FGF21 exerts its metabolic effects in part by activating the sympathetic nervous system, which induces thermogenesis in BAT and browning of white adipose tissue (4, 5). We recently showed that FGF21 also acts directly on the locus coeruleus (LC) in mice to stimulate the noradrenergic nervous system (6), which can also modulate thermogenesis (7, 8). These findings suggest the possibility of an additional pathway whereby FGF21 could exert thermoregulatory effects.

Circulating FGF21 is induced in mice by bacterial inflammation (9, 10) and in human patients infected with hepatitis B (11), hepatitis C (12), HIV (13–15), or severe acute respiratory syndrome coronavirus 2 (16). In this study, we show that FGF21 is induced in mice by influenza infection and protects against the resulting anorexia, weight loss, and hypothermia. We further show that FGF21 acts directly on the noradrenergic nervous system to elicit these protective effects. Our findings suggest that FGF21 may prove useful as a drug for treating influenza infection.

## Results

### Influenza Infection Induces FGF21.

**Fig. 1.**
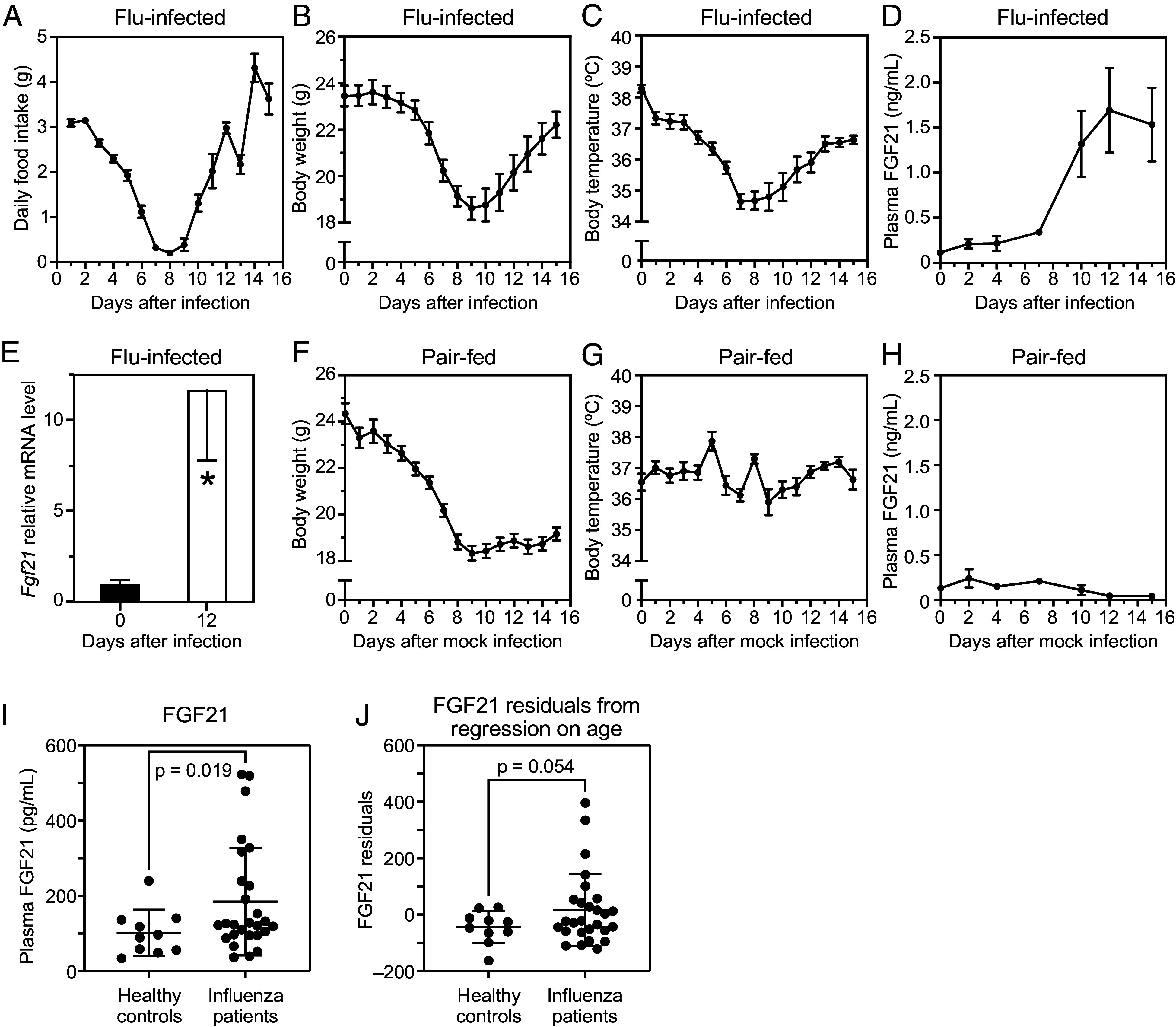
Influenza infection causes decreased body weight, body temperature, food intake, and elevated plasma FGF21. (*A*) Daily food intake, (*B*) body weight, (*C*) body temperature (n = 9/group), and (*D*) plasma FGF21 concentration (n = 4 to 7/group) in WT mice after influenza infection. (*E*) *Fgf21* mRNA expression in liver analyzed by qPCR either 0 or 12 d after influenza infection (n = 5 to 6/group). (*F*) Body weight, (*G*) body temperature (n = 7/group), and (*H*) plasma FGF21 concentration (n = 3 to 7/group) in WT mice intranasally inoculated with phosphate-buffered saline (PBS) and then food restricted to the daily consumption of influenza-infected mice (pair-feeding). (*I* and *J*) FGF21 serum levels in healthy and influenza human patients before and after correcting for age-related effects. (*I*) Plasma FGF21 in 27 influenza patients at the time of emergency room blood draw and in 10 healthy controls. (*J*) Residuals from the regression of FGF21 on age from Panel *I*. *P*-value by Welch’s *t* test, where * indicates *P* < 0.05. Data are shown as the mean ± SEM.

As expected, infection of wild-type (WT) mice with influenza caused decreases in daily food intake, body weight, and body temperature that reached their nadir 7 to 9 d postinfection (Fig. 1 *A*–*C*). Notably, improvements in these parameters coincided with a large increase in plasma FGF21 concentrations (Fig. 1*D*) and a corresponding increase in hepatic *Fgf21* messenger RNA (mRNA) (Fig. 1*E*). To determine whether the induction of FGF21 was secondary to decreased food intake, a pair-feeding study was performed in mock-infected mice. While body weight decreased comparably in influenza-infected and uninfected pair-fed mice (compare Fig. 1 *B* and *F*), there was neither a decrease in body temperature nor an induction of FGF21 in the pair-fed group (Fig. 1 *G* and *H*). Importantly, we found that serum FGF21 concentrations were also increased in influenza-infected humans compared to healthy controls (Fig. 1*I*). Although age also drives increased serum FGF21 levels (17), plotting the residuals from the regression of FGF21 on age still demonstrated that influenza infection was associated with increased serum FGF21 concentrations (Fig. 1*J*). Using a heteroskedasticity-robust multiple regression method, we found that influenza infection status was independently associated with FGF21 levels after accounting for age (*SI Appendix*, Table S1). Thus, influenza-induced changes in body temperature and circulating FGF21 concentrations in mice are caused by the viral infection not by reduced food intake or body weight, and influenza induces FGF21 in both mice and humans.

### Endogenous FGF21 Protects against Influenza Infection via the Noradrenergic Nervous System.

**Fig. 2.**
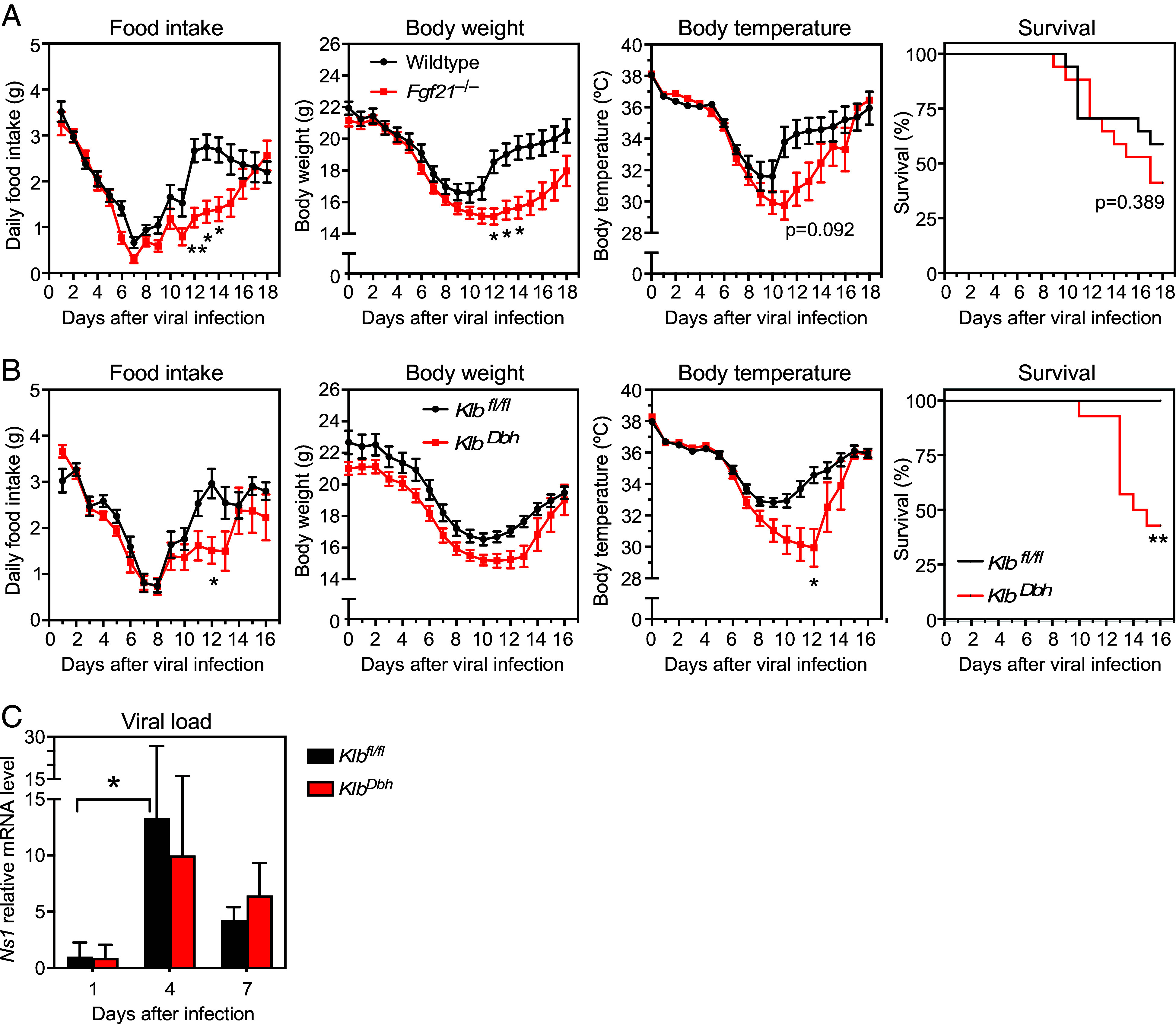
FGF21 acts on noradrenergic neurons to induce the recovery of body weight, body temperature, and food intake after influenza infection. (*A*) Daily food intake, body weight, body temperature, and survival in WT and *Fgf21*^–/–^ mice after influenza infection (n = 17/group). (*B*) Daily food intake, body weight, body temperature, and survival in *Klb^fl/fl^* and *Klb^Dbh^* mice after influenza infection (n = 11 to 14/group). (*C*) Viral load in *Klb^fl/fl^* and *Klb^Dbh^* mice analyzed by mRNA expression of influenza *Ns1* in infected lungs (n = 3 to 5/group). Data are shown as the mean ± SEM. *P < 0.05, **P < 0.01, ***P < 0.001.

To test whether endogenous FGF21 protects against influenza, groups of WT and *Fgf21^–/–^* mice were infected with the virus. While the decreases in daily food intake, body weight, and body temperature were similar between the two genotypes over the first 5 to 7 d, *Fgf21^–/–^* mice showed a more pronounced reduction in these parameters and a slower recovery than WT mice after that (Fig. 2*A*). There was no difference in survival between genotypes (Fig. 2*A*).

Since the noradrenergic nervous system is involved in the febrile response (18), we performed this same experiment in control *Klb^fl/fl^* and *Klb^Dbh^* mice, in which FGF21’s obligate coreceptor KLB was selectively eliminated in noradrenergic neurons. Importantly, the *Klb^Dbh^* mice showed the same delayed recovery in daily food intake, body weight, and body temperature following influenza infection as *Fgf21^–/–^* mice (Fig. 2*B*). There was also a striking decrease in the survival of *Klb^Dbh^* mice compared to control *Klb^fl/fl^* mice (Fig. 2*B*). The reason for the increased survival of the control *Klb^fl/fl^* mice in Fig. 2*B* compared to WT mice in Fig. 2*A* is unclear but was seen in two independent experiments. There were no differences between control and *Klb^Dbh^* mice in viral load in lung at days 4 and 7 postinfection (Fig. 2*C*) and analysis of a large panel of cytokines in plasma revealed minor changes in IL-1α, IL-5, GM-CSF, Eotaxin, and KC that reached significance at only single time points each postinfection (*SI Appendix*, Fig. S1). Taken together, these data show that FGF21 acts directly on noradrenergic neurons to protect against influenza infection without affecting viral replication.

### Pharmacologic FGF21 Acts Directly on Noradrenergic Neurons to Increase Energy Expenditure.

**Fig. 3.**
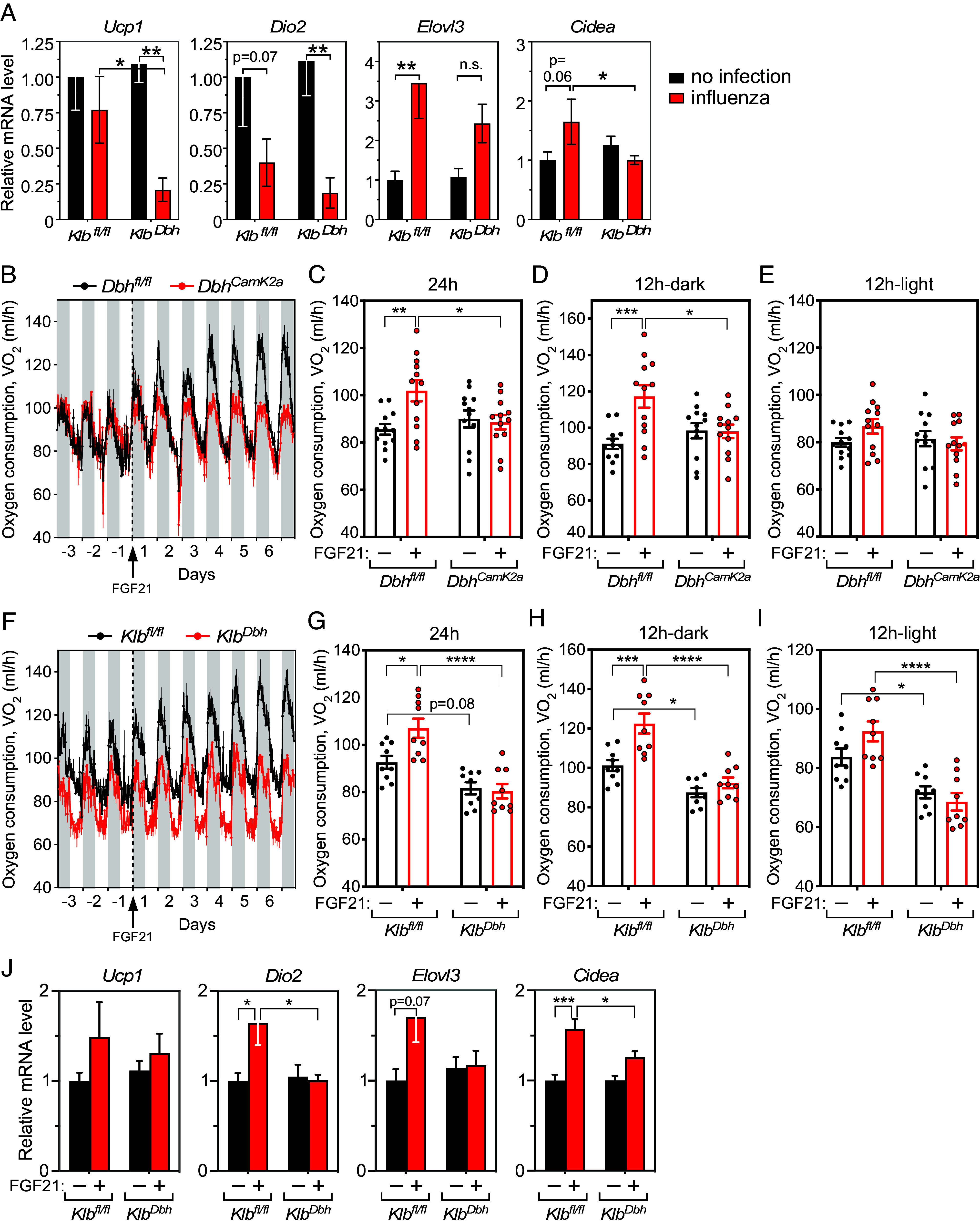
FGF21 increases systemic energy expenditure by acting through the noradrenergic nervous system. (*A*) The mRNA expression of *Ucp1*, *Dio2*, *Elovl3*, and *Cidea* in BAT in *Klb^fl/fl^* and *Klb^Dbh^* mice analyzed by qPCR at day 12 post influenza infection (n = 6 to 7/group). (*B*) Oxygen consumption of *Dbh^fl/fl^* and *Dbh^CamK2a^* mice treated with vehicle (once/day) for 3 d followed by FGF21 (1 mg/kg/d, i.p.) for 6.5 d (n = 12/group). The arrow and dotted line indicate the beginning of FGF21 treatment. Gray bars indicate the nighttime. (*C*–*E*) Average oxygen consumption recorded during the whole day (*C*), the nighttime (*D*), and the daytime (*E*) in *Dbh^fl/fl^* and *Dbh^CamK2a^* mice treated with vehicle or FGF21 (n = 12/group). Measurements for (*C*–*E*) were taken on day –2 (for vehicle) and day 6 (for FGF21). (*F*) Oxygen consumption of *Klb^fl/fl^* and *Klb^Dbh^* mice treated with vehicle (once/day) for 3 d followed by FGF21 (1 mg/kg/d, i.p.) for 6.5 d (n = 9/group). The arrow and dotted line indicate the beginning of FGF21 treatment. Gray bars indicate the nighttime. (*G*–*I*) Average oxygen consumption recorded during the whole day (*G*), the nighttime (*H*), and the daytime (*I*) in *Klb^fl/fl^* and *Klb^Dbh^* mice treated with vehicle or FGF21 (n = 9/group). Measurements for (*G*–*I*) were taken on day –2 (vehicle) and day 6 (FGF21). (*J*) The mRNA expression of *Ucp1*, *Dio2*, *Elovl3*, and *Cidea* in BAT in vehicle (black bars) or FGF21 (red bars)-treated *Klb^fl/fl^* and *Klb^Dbh^* mice analyzed by qPCR (n = 6/group). Data are shown as the mean ± SEM. *P < 0.05, **P < 0.01, ***P < 0.001, ****P < 0.0001.

Analysis of thermogenic gene expression in BAT, which contributes to the febrile response (19–21), revealed significant decreases in *Ucp1* and *Cidea* mRNA in influenza-infected *Klb^Dbh^* mice compared to *Klb^fl/fl^* control mice at 12 d post infection (Fig. 3*A*) and similar trends for *Dio2* and *Elovl3*, suggesting a possible mechanism for FGF21’s effect on body temperature. To examine whether FGF21 stimulates energy expenditure via the noradrenergic nervous system, we measured oxygen consumption in either control *Dbh^fl/fl^* or *Dbh^Camk2a^* mice, in which dopamine β-hydroxylase (DBH), and thus norepinephrine synthesis, was selectively eliminated in *Camk2a*^+^ neurons (6). *Dbh^fl/fl^* and *Dbh^Camk2a^* mice were first administered vehicle for 3 d to establish baseline oxygen consumption, followed by 6.5 d of FGF21 administration (1 mg/kg, i.p., q.d.). There was no difference in oxygen consumption between the two genotypes under baseline conditions (Fig. 3 *B* and *C*). However, FGF21 administration increased oxygen consumption significantly in control mice but not in *Dbh^Camk2a^* mice (Fig. 3 *B* and *C*). The pharmacologic effect of FGF21 was more pronounced in the dark cycle, when mice are active and feeding, than in the light cycle (Fig. 3 *D* and *E*). Thus, the effect of exogenous FGF21 on oxygen consumption requires norepinephrine synthesis in neurons.

We next examined whether FGF21 increases energy expenditure by acting directly on noradrenergic neurons. Oxygen consumption was measured in control *Klb^fl/fl^* or *Klb^Dbh^* mice, which lack FGF21’s obligate coreceptor KLB in *Dbh*^+^ neurons (6). Interestingly, basal energy expenditure was slightly decreased in *Klb^Dbh^* mice (Fig. 3 *F* and *G*). FGF21 administration significantly increased oxygen consumption in control but not *Klb^Dbh^* mice (Fig. 3 *F* and *G*), and this effect was observed in both the dark and light cycles (Fig. 3 *F*, *H*, and *I*). Accordingly, FGF21 significantly increased the expression of thermogenic genes *Dio2* and *Cidea* and caused similar trends for *Ucp1* and *Elovl3* in BAT from control but not *Klb^Dbh^* mice (Fig. 3*J*). FGF21 did not affect the respiratory exchange ratio (RER) in lean control mice (*SI Appendix*, Fig. S2 *A* and *B*), demonstrating that it stimulates energy expenditure without changing energy substrate utilization.

**Fig. 4.**
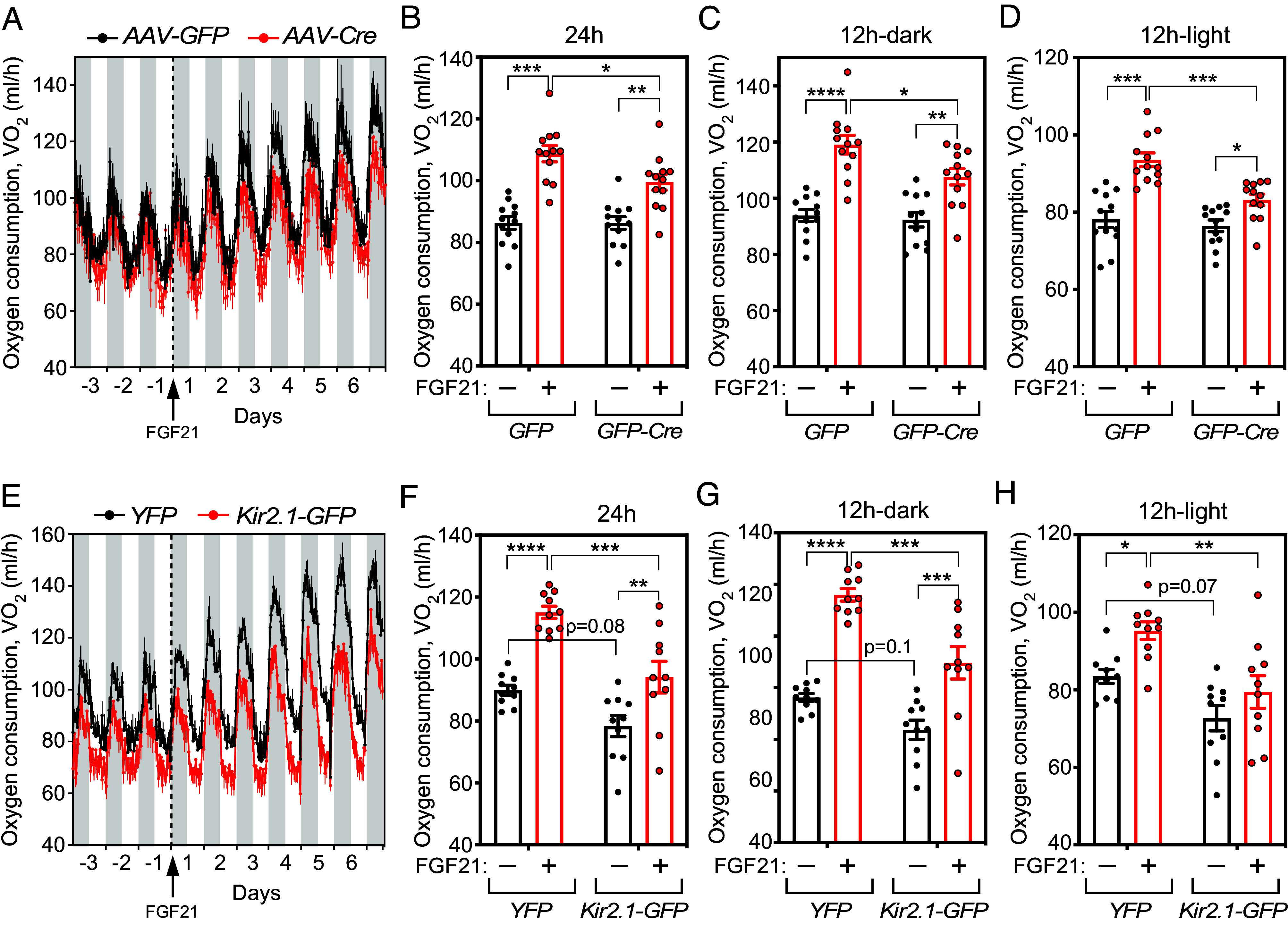
FGF21 regulates energy expenditure by acting on *Klb*-expressing neurons in the LC area. (*A*) Oxygen consumption of *Klb^fl/fl^* mice injected with green fluorescent protein (GFP) or GFP-Cre virus and subsequently treated with vehicle (once/day) for 3 d followed by FGF21 (1 mg/kg/d, i.p.) for 6.5 d (n = 12/group). The arrow and dotted line indicate the beginning of FGF21 treatment. Gray bars indicate the nighttime. (*B*–*D*) Average oxygen consumption recorded during the whole day (*B*), the nighttime (*C*), and the daytime (*D*) in *Klb^fl/fl^* mice injected with GFP or GFP-Cre virus and treated with vehicle or FGF21 (n = 12/group). Measurements for (*B*–*D*) were taken on day –2 (vehicle) and day 6 (FGF21). (*E*) Oxygen consumption of *Klb*-Cre mice injected with Ef1a-DIO-EYFP or hSyn-FLEx-Kir2.1-2A-GFP virus and subsequently treated with vehicle (once/day) for 3 d, followed by FGF21 (1 mg/kg/d, i.p.) for 6.5 d (n = 10/group). The arrow indicates the beginning of FGF21 treatment. Gray bars indicate the nighttime. (*F*–*H*) Average oxygen consumption recorded during the whole day (*F*), the nighttime (*G*), and the daytime (*H*) in *Klb*-Cre mice injected with Ef1a-DIO-EYFP or hSyn-FLEx-Kir2.1-2A-GFP virus and treated with vehicle or FGF21 (n = 10/group). Measurements for (*B*–*D*) were taken on day –2 (vehicle) and day 6 (FGF21). Data are shown as the mean ± SEM. *P < 0.05, **P < 0.01, ***P < 0.001, ****P < 0.0001.

Most noradrenergic neurons are located in the LC, where we previously showed that FGF21 acts to accelerate sobering in alcohol intoxicated mice (6). We took two experimental approaches to address whether FGF21 stimulates energy expenditure by acting on neurons in the LC region. First, *Klb^fl/fl^* mice were injected bilaterally with either AAV-GFP-Cre or a control AAV-GFP virus directly into the LC region (*SI Appendix*, Fig. S3*A*). Mice injected with AAV-GFP-Cre had a significant reduction in oxygen consumption in response to FGF21 administration compared to mice injected with control virus (Fig. 4 *A*–*D*).

In a second approach, we selectively inhibited *Klb*-expressing neurons in the LC region using the inwardly rectifying potassium channel Kir2.1 (22). To enable these experiments, we generated a *Klb*-Cre mouse line which, as expected based on our previous findings (6), expresses Cre in noradrenergic neurons in the LC (*SI Appendix,* Fig. S3*B*). *Klb*-Cre mice were injected bilaterally into the LC region with either hSyn-FLEx-Kir2.1-2A-GFP or Ef1a-DIO-EYFP as a control. In agreement with our results with *Klb^Dbh^* mice, Kir2.1 expression caused a trend toward decreased basal oxygen consumption and significantly blunted induction of oxygen consumption by FGF21 (Fig. 4 *E*–*H*). We conclude that KLB in noradrenergic neurons, including *Klb*^+^ neurons in the LC region, modulates FGF21-induced energy expenditure.

### Pharmacologic FGF21 Protects against Influenza Infection.

**Fig. 5.**
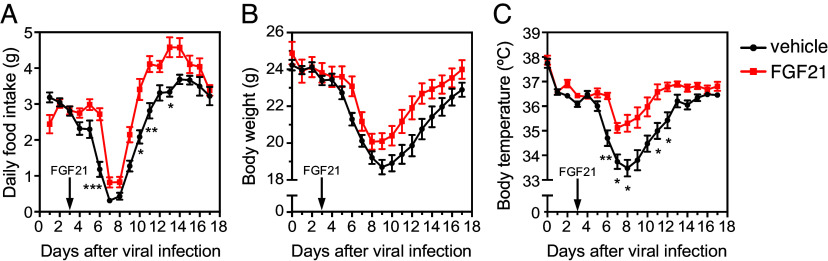
Pharmacologic FGF21 promotes recovery of body weight, body temperature, and food intake after influenza infection. (*A*) Daily food intake, (*B*) body weight, and (*C*) body temperature in WT mice injected with vehicle (once/day) or FGF21 (1 mg/kg/d, i.p.) after influenza infection (n = 12 to 16/group). The arrow indicates the beginning of the FGF21 treatment on day 3 after influenza infection. Data are shown as the mean ± SEM. *P < 0.05, **P < 0.01, ***P < 0.001.

Finally, we examined whether pharmacologic FGF21 protects against influenza virus infection. WT mice were administered FGF21 or vehicle daily by i.p. injection starting 3 d postinfection. Notably, FGF21 protected against the anorexia, weight loss, and hypothermia in infected mice (Fig. 5 *A*–*C*). Thus, pharmacologic administration of FGF21 promotes recovery from the pathological effects of influenza infection.

## Discussion

The ability of animals to survive infection requires both disease resistance, which involves pathogen clearance, and disease tolerance, which minimizes the detrimental effects of infection on host health without directly impacting the pathogen burden (23). In this report, we show that FGF21 is involved in adaptive tolerance to influenza infection in mice. FGF21 is induced 8 to 10 d after infection and correlates with the recovery phase. Mice lacking FGF21 eat less, lose more body weight, and are more hypothermic compared to WT mice. Conversely, daily administration of pharmacologic FGF21 to mice starting 3 d after infection protects against the anorexia, weight loss, and hypothermia. Furthermore, influenza infection increases serum FGF21 concentrations in humans. Together, these findings suggest that FGF21 may be beneficial as a drug for treating influenza infection.

We further show that all of FGF21’s protective effects against influenza, including its effect on body temperature, require it to act directly on noradrenergic neurons. Previous studies demonstrated that FGF21 is involved in maintaining core body temperature during cold exposure. FGF21 was shown to be induced by cold in both white adipose tissue, where it acts through an autocrine mechanism to promote browning (24, 25), and in liver, which secretes it into the blood to stimulate sympathetic outflow from the brain to BAT (26). Our current findings support the existence of an alternate noradrenergic nervous system-BAT pathway that mediates circulating FGF21’s beneficial effect on body temperature during influenza infection. Thermogenic gene expression was dysregulated in the BAT of *Klb^Dbh^* mice during infection, and pharmacologic FGF21 stimulated energy expenditure in healthy mice by acting directly on noradrenergic neurons, including *Klb*^+^ neurons in the LC region. Accordingly, pharmacologic administration of FGF21 starting at 3 d postinoculation protected against the hypothermia caused by influenza. These results are consistent with previous work showing that influenza infection rewires energy metabolism and induces browning of white adipocytes (27) and support the conclusion that FGF21 may be mediating this effect as a metabolic adaption to infection.

Previous studies performed in rats and guinea pigs have linked the noradrenergic nervous system to body temperature via the febrile response. Both electrolytic and chemical lesions of the LC attenuated LPS-induced fever (7). Moreover, both lipopolysaccharide (LPS) and interleukin-1β (IL-1β), which is also a pyrogen, were shown to increase norepinephrine concentrations in the hypothalamic preoptic area, which regulates body temperature (28, 29). Norepinephrine, in turn, has been shown to trigger the release of the fever mediator, PGE_2_, in the preoptic area, which can stimulate BAT-mediated thermogenesis (30, 31). Some LC neurons are also connected multisynaptically to BAT and modulate its thermogenic activity through a more direct mechanism (8). Additional studies will be required to elucidate precisely how the FGF21 pathway protects against the hypothermia as well as the anorexia and weight loss caused by influenza infection.

FGF21 has also been shown to be induced by and to protect against bacterial sepsis. *Fgf21^–/–^* mice were much more susceptible than WT littermates to the hypothermia and mortality caused by either LPS injection or cecal ligation and puncture (10). In the latter model, there was no difference in bacterial load between control *Fgf21^fl/fl^* and *Fgf21^–/–^* mice. We conclude that FGF21 regulates tolerance to both bacterial and viral sepsis and that it may be useful pharmacologically for treating a range of bacterial and viral infections.

## Materials and Methods

### Mouse Studies.

All procedures and use of mice were approved by the Institutional Animal Care and Use Committees of University of Texas Southwestern Medical Center. Mice were housed in a temperature-controlled environment with 12-h light–dark cycles. Housing rooms were maintained at 22 to 23 °C. Mice were maintained on a standard rodent chow diet. Age-matched 3- to 5-mo-old male mice were used for all experiments except for the influenza infection experiment. *Klb^Dbh^* mice were generated by crossing *Dbh*-*Cre* mice (Jackson Laboratory, Stock No: 033951) with *Klb^fl/fl^* mice. *Dbh^Camk2a^* mice were generated by crossing *Camk2a*-*Cre* mice with *Dbh^fl/fl^* mice (6). Recombinant human FGF21 protein was provided by Novo Nordisk and administered by i.p. injection at a dose of 1 mg/kg/d. For the influenza infection experiment, virus was administered intranasally in age-matched 8- to 12-wk-old male mice. C57BL/6J WT mice were purchased from the Jackson Laboratory (Stock No. 000664). The generation of *Fgf21* KO mice was previously described (32).

### Influenza Viral Infection.

The preparation of the cell supernatant containing influenza A virus was previously described (33). All mice were anesthetized with isoflurane (5% for induction and 2% for maintenance) prior to intranasal inoculation with 125 or 250 plaque-forming units in 50 μL PBS. Body weight, body temperature, daily food intake, and mortality were recorded every day for 15 to 18 d postinfection. Core body temperature was measured by rectal thermometer.

### Stereotaxic Surgery.

Mice were anesthetized with 1.5% isoflurane and placed on a stereotaxic frame (David Kopf Instruments). Heating pads were used throughout the duration of the surgery to maintain a stable body temperature. Eye ointment was applied to keep the eyes from drying. After the skull was exposed and leveled in the horizontal plane, small holes were drilled for virus injections. Adeno-associated virus (AAV) was bilaterally injected into the LC (AP, –5.40 mm; ML, ± 0.90 mm; DV, –4.10 mm) using a NanoFil syringe (World Precision Instruments, 10 µL) with NanoFil injection needle (34G, World Precision Instruments, NF34BV-2) and microinjection syringe pump (World Precision Instruments, UMP3T-1). A total volume of 300 nL of the virus was injected at a rate of 50 nL/min. The injection needle was left in the brain for 5 min before it was removed to allow the virus to diffuse into the brain tissue. AAV particles were prepared by the University of North Carolina at Chapel Hill (UNC) Vector Core. AAV vectors used for stereotaxic injections included AAV8-hSyn-GFP (UNC Vector Core), AAV8-hSyn-GFP-Cre (UNC Vector Core), AAV9-Ef1a-DIO-EYFP (Addgene #27056), and AAV8-hSyn-FLEx-loxp-Kir2.1-2A-GFP (Addgene #161574). Mice were allowed to recover for 3 to 4 wk postsurgery to allow for proper viral spread and plasmid vector expression. The AAV injection sites were verified by checking the expression of the fluorescent reporters in the brain of each mouse, and mice in which the injections missed the desired targets were excluded from the experimental results.

### Oxygen Consumption and RER Measurement.

Metabolic cage studies were performed by the University of Texas Southwestern Medical Center Metabolic Phenotyping Core at 22 to 23 °C. Indirect calorimetry using LabMaster metabolic cages (TSE systems) was performed to measure oxygen consumption and RER. Body composition was measured using an EchoMRI-100 body composition analyzer. Mice were given vehicle or FGF21 (1 mg/kg, i.p.) once every day at the same time (4:00 p.m., 2 h before lights off) during the recording period.

### Murine Plasma FGF21 and Cytokine Measurements.

Blood was collected into ethylenediaminetetraacetic acid-coated tubes (Sarstedt). Plasma was separated by centrifugation at 3,000 rpm for 30 min at 4 °C. Plasma FGF21 was measured using ELISA (R&D Systems). Plasma cytokines were measured using Mouse Cytokine 23-plex Assay (Bio-Rad #M60009RDPD) and Procartaplex assay (ThermoFisher #EPX02A-22187-901).

### Human Plasma FGF21 Measurements.

Subjects were recruited after presentation to the emergency department at New York Presbyterian Hospital/Weill Cornell Medicine with a diagnosis of influenza between December 2019 and February 2020. The diagnosis of influenza was made by respiratory pathogen PCR of a nasopharyngeal swab. Plasma from influenza patients was collected during their emergency department stay, prior to hospital admission or discharge from the emergency department, and stored at −80 °C. Plasma from 10 healthy controls was collected from individuals recruited from a waiting room of a primary care clinic at Weill Cornell Medicine and stored at −80 °C. Of 54 influenza patients with plasma available, 26 were excluded due to age younger than available controls (<40 y), active cancer, or chronic kidney disease. Plasma from the remaining 28 influenza patients and 10 healthy controls was used for sandwich ELISA using the BioVendor FGF21 human ELISA kit according to the manufacturer’s instructions. One influenza subject was subsequently excluded because the FGF21 level was out of the quantifiable range of the ELISA. The FGF21 level in influenza patients and healthy controls was compared by 1) Welch’s *t* test and 2) with adjustment for age using multiple regression with heteroskedasticity-robust SE in R [H5 estimator; (34)]. The study was approved by the institutional review board of Weill Cornell Medicine under protocol number 24-04027385.

### Generation of *Klb*-Cre Mice.

*Klb*-Cre mice, in which a P2A-Cre coding sequence was inserted in-frame after exon 5 of the *Klb* gene, were generated using a CRISPR/Cas-9 gene editing strategy. Briefly, the *Klb*-Cre transgenic plasmid was generated using KLB-T plasmid (35) as a backbone and the tdTomato sequence was replaced with the P2A-Cre sequence. The crRNA (5′-CAUAGUUUCAAGAUUCACUCGUUUUAGAGCUAUGCU-3′) and tracrRNA were purchased from Integrated DNA Technologies, Inc. The crRNA, tracrRNA, and Cas9 protein were coinjected into fertilized oocytes from C57BL/6N female mice at the University of Texas Southwestern Medical Center Transgenic Core. Founder mice were bred with C57BL/6J mice, and progeny were screened for the correct transgene by PCR and sequencing. We used 5′-CTCCCCGCTGAGAACAGAAG-3′, 5′-TTGCGAACCTCATCACTCGT-3′, and 5′-TACCAGTACATGGAGCCGGA-3′ primers for mice genotyping.

### In Situ Hybridization and Immunofluorescence.

The *Klb*-Cre mice were anesthetized with 1.5% isoflurane and transcardially perfused first with PBS followed by 10% neutral buffered formalin (NBF). The brains were then harvested and incubated in 10% NBF for at least 24 h at 4 °C. Fixed brains were washed with PBS briefly and stored in PBS containing 0.01% NaN3. Brain sections were embedded in 4% low-melting agarose and sectioned in a Leica VT1000S vibratome at a section thickness of 25 µm. Free-floating brain sections were collected in PBS and then treated with hydrogen peroxide for 10 min. After rinsing with distilled water, the sections were mounted and desiccated overnight at room temperature. In situ hybridization was performed by the University of Texas Southwestern Medical Center Metabolic Phenotyping Core using an RNAscope Multiplex Fluorescent Detection Kit (Cat No. 323110) and *Klb* (Cat No. 415221-C2, Mm-Klb-C2) and Cre (Cat No. 312281) probes purchased from Advanced Cell Diagnostics. Hybridized brain sections were sequentially incubated with amplification reagents and Opal 570 and 690 dyes (Akoya Biosciences, 1:1,500 dilution). After the in situ hybridization, sections were incubated with tyrosine hydroxylase (TH) primary antibody (Aves Labs TYH, 1:1,000 dilution) overnight at 4 °C in dark. Sections were washed three times in PBS followed by incubation with Alexa Fluor-conjugated goat antichicken IgY secondary antibody (Invitrogen, 1:500 dilution) and DAPI (Thermo Fisher Scientific, 1:5,000 dilution) for 2 h at room temperature in dark. Both primary and secondary antibodies were diluted in blocking buffer (5% normal goat serum, 1% bovine serum albumin (BSA), 0.3% Triton X-100 in PBS). For immunostaining of GFP in AAV8-hSyn-GFP and AAV8-hSyn-GFP-Cre, brains were sectioned at a section thickness of 50 µm. Free-floating brain sections were incubated in blocking buffer (5% normal goat serum, 1% BSA, 0.3% Triton X-100 in PBS) for 1 h at room temperature followed by incubation in primary antibody for TH (Aves Labs TYH, 1:1,000 dilution) and GFP (Abcam ab290, 1:500 dilution) at 4 °C for 1 d. Brain sections were washed three times in PBS (10 min/wash) and incubated in secondary antibody (Alexa Fluor-conjugated goat antichicken IgY and goat antirabbit IgG, 1:500 dilution) and DAPI (Thermo Fisher Scientific, 1:5,000 dilution) for 2 h at room temperature in dark. Brain sections were then washed three times in PBS (10 min/wash) and mounted. Images were taken using a Zeiss LSM780 confocal microscope and were processed using Zeiss ZEN software.

### Real-Time qPCR Analysis.

Total RNA was isolated from tissue using RNA-STAT60 reagent (Tel-Test CS502), and cDNA was synthesized using the High-Capacity Reverse Transcription Kit (Applied Biosystems). QPCR was performed with an Applied Biosystems 7900HT Sequence Detection System using the ΔΔCt method. For viral load measurement in the lung, the mRNA level of influenza A virus *NS1* (nonstructural protein 1) gene was measured by QPCR as previously described (36).

### Statistical Analysis.

All data were presented as mean ± SEM and analyzed by two-way ANOVA. Differences were considered statistically significant at *P* < 0.05.

## Supplementary Material

Appendix 01 (PDF)

## Data Availability

All study data are included in the article and/or *SI Appendix*.
