# Supplementary material for: FGF21 acting on the noradrenergic nervous system protects against influenza virus infection
Source: Proc Natl Acad Sci U S A. 2025 Sep 25;122(39):e2522045122. doi: 10.1073/pnas.2522045122 (PMC12501196; doi:10.1073/pnas.2522045122)
Supplement: Supplementary file 1 — Appendix 01 (PDF) [file pnas.2522045122.sapp.pdf]

PNAS

Supporting Information for

FGF21 Acting on the Noradrenergic Nervous System Protects Against Influenza Virus Infection

Wei Fan, Yuan Zhang, Laurent Gautron, David G. Thomas, Heather W. Stout-Delgado, Edward J. Schenck, Tadiwanashe Gwatiringa, Kartik N. Rajagopalan, David J. Mangelsdorf, Steven A. Kliewer

Corresponding authors: Kartik N. Rajagopalan, Steven A. Kliewer
Email: kartik.rajagopalan@utsouthwestern.edu, steven.kliewer@utsouthwestern.edu

This PDF file includes:

Figures S1-S3
Table S1

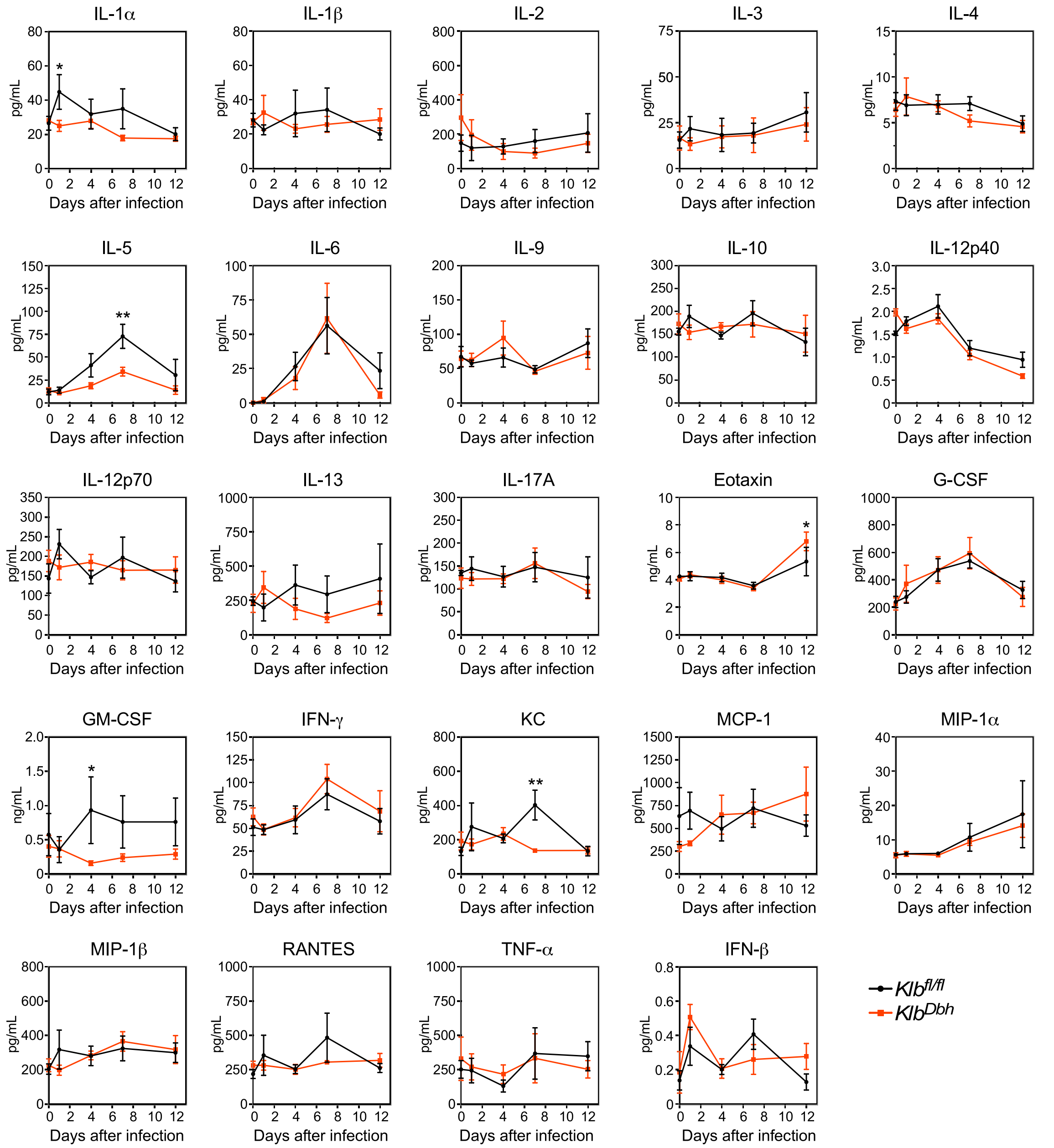

Fig. S1. Multiple plasma cytokine levels show little to no significant differences between influenza infected $Klb^{fl/fl}$ and Klb^{Dbh} mice. Plasma cytokine levels after influenza infection were assayed as described in methods (n=4-8/group). Data are shown as the mean ± SEM. *$p < 0.05$, **$p < 0.01$.

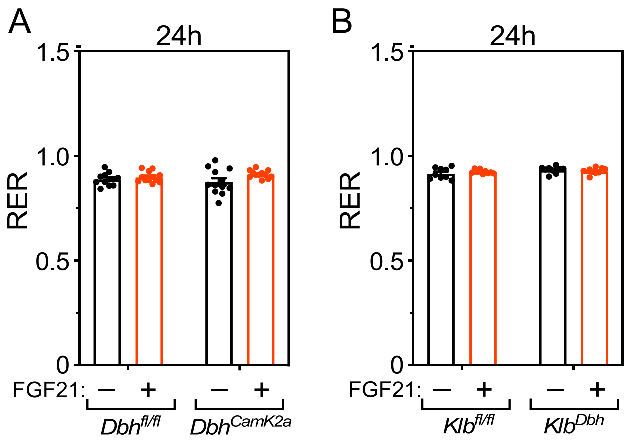

Fig. S2. Respiratory exchange ratio (RER) in control, *Dbh*^{Camk2a}, and *Klb*^{Dbh} mice. (A and B) Average RER levels recorded during the whole day in *Dbh*$^{fl/fl}$ and *Dbh*^{CamK2a} mice (n=12/group) (A), *Klb*$^{fl/fl}$ and *Klb*^{Dbh} mice (n=9/group). (B) Mice were treated with vehicle (once/day) for 3 days followed by FGF21 (1mg/kg/day, i.p.) for 6.5 days. Measurements were taken on day –2 (for vehicle) and day 6 (for FGF21). Data are shown as the mean ± SEM. These RER values were calculated from the same 24h experiments shown in Fig. 3 C and G.

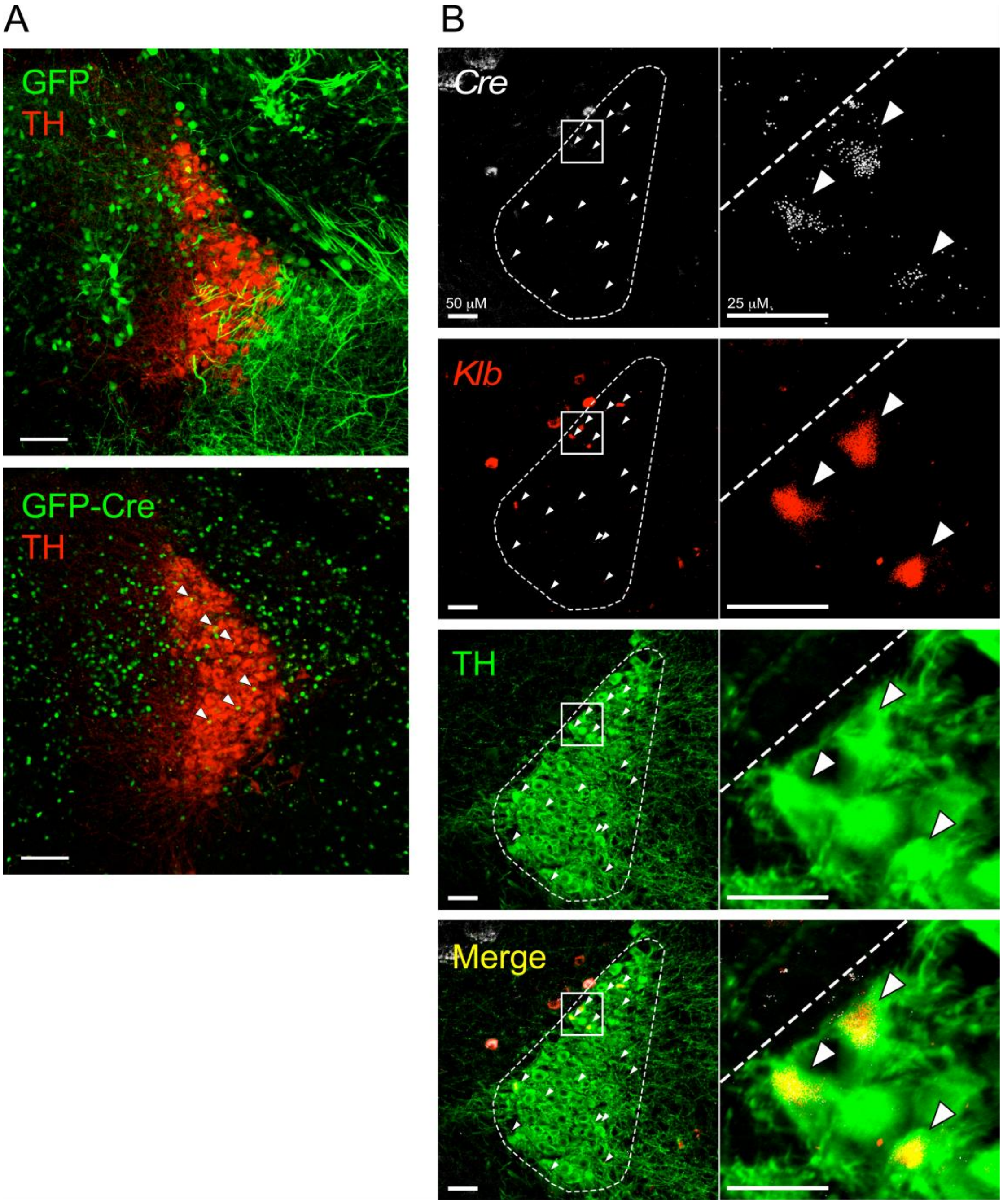

Fig. S3. Validation of Cre drivers targeting the locus coeruleus. (A) GFP and GFP-Cre expression in the locus coeruleus area of *Klb*$^{fl/fl}$ mice injected with AAV8-hSyn-GFP and AAV8-hSyn-GFP-Cre virus. Immunostaining of tyrosine hydroxylase (TH, red) and GFP (green) of *Klb*$^{fl/fl}$ mice injected into the locus coeruleus area with an AAV expressing either GFP (top panel) or GFP-Cre (bottom panel). Note that GFP alone localizes to the cytosol, whereas the GFP-Cre fusion localizes to the nucleus. Arrowheads point to TH neurons that co-express *Cre* in the

bottom panel. Scale bars represent 100 μM. (B) *Klb* and *Cre* are co-expressed in the locus coeruleus in *Klb*-Cre mice. The four panels from top to bottom show in situ hybridizations of *Cre* mRNA (white) and *Klb* mRNA (red), immunostaining for tyrosine hydroxylase (TH, green), and the merge (yellow) of the three, respectively. Arrowheads point to TH neurons that co-express *Cre* and *Klb*. The white dotted lines outline the locus coeruleus. Two different magnifications are shown, with the boxed area in the left panels expanded in the right panels. Scale bars represent 50 μM in left panels and 25 μM in right panels.

Table S1. Multiple regression analysis for influenza infection status and age on serum FGF21 concentrations.

	Regression Coefficient	95% Confidence Interval	Regression p-value
**Age**	3.62	1.44-5.79	0.003
**Influenza**	62.5	2.8-122.2	0.048
